# Lhx8 interacts with a novel germ cell-specific nuclear factor containing an Nbl1 domain in rainbow trout (*Oncorhynchus mykiss*)

**DOI:** 10.1371/journal.pone.0170760

**Published:** 2017-02-02

**Authors:** Liyuan Fu, Prasanthi P. Koganti, Jian Wang, Lei Wang, Cheng-Lun Wang, Jianbo Yao

**Affiliations:** Division of Animal and Nutritional Sciences, West Virginia University, Morgantown, West Virginia, United States of America; Institut National de la Recherche Agronomique, FRANCE

## Abstract

Lhx8 is an important transcription factor that is preferentially expressed in germ cells. *Lhx8* null mice are infertile due to lack of oocytes and impairment of the transition from primordial follicles to primary follicles. *Lhx8* deficiency also affects the expression of many important oocyte-specific genes. In this study, we report the characterization of rainbow trout *lhx8* genes and identification of a novel germ cell-specific nuclear factor that interacts with Lhx8. Two *lhx8* genes, *lhx8a* and *lhx8b*, were identified, encoding proteins of 344 and 361 amino acids, respectively. The two proteins share 83% sequence identity and both transcripts are specifically expressed in the ovary. Quantitative real time PCR analysis demonstrated that both genes are expressed highly in pre-vitellogenic ovaries as well as in early stage embryos. Using a yeast two-hybrid screening system, a novel protein (Borealin-2) interacting with Lhx8 was identified. The interaction between either Lhx8a or Lhx8b and Borealin-2 was further confirmed by a bimolecular fluorescence complementation (BiFC) assay. Borealin-2 is a protein of 255 amino acids containing an Nbl1 domain, and its mRNA expression is restricted to the ovary and testis. A GFP reporter assay revealed that Borealin-2 is a nuclear protein. Collectively, results indicate that both Lhx8a and Lhx8b function through interaction with Borealin-2, which may play an important role during oogenesis and early embryogenesis in rainbow trout.

## Introduction

Through gene knockout studies in mouse, the functional roles of many oocyte-specific factors during folliculogenesis and early embryonic development have been revealed [1]. A number of key oocyte-specific nuclear factors known to be vital in follicular development are transcription factors, which include factor in the germline alpha (Figla) [2], newborn ovary homeobox (Nobox) [3], and LIM homeobox 8 (Lhx8) [4]. However, our understanding of the regulatory mechanisms of these important transcription factors in oocyte and follicular development is far from complete, particularly in species like fish.

Rainbow trout is a major fish species cultivated widely in the world for food production and sport fisheries. It is also utilized as a model species in research [5]. Understanding the functional contribution of oocyte-specific genes to egg quality will help us develop biomarkers for prediction of egg quality in rainbow trout. Our previous studies have resulted in the discovery of a number of novel oocyte-specific genes in rainbow trout, which include *oorp-t* [6], *rtgst-1* [7], *fbxoo* [8] and *knpa7* [9].

Lhx8 is a member of the LIM-homeobox transcription factor family and its transcript is detected in oocytes of follicles of various stages in mouse ovary [4]. Lhx8 protein is located in the nucleus of oocytes and is increasingly expressed during primordial follicle activation [10]. *Lhx8* null mice are infertile due to lack of oocytes and impairment of the transition from primordial follicles to primary follicles [10] Conditional deletion of *Lhx8* from oocytes of primary follicles leads to primary follicle death and severe loss of the secondary and antral follicles.[11]. Lhx8 deficiency affects the expression of many important oocyte-expressed genes such as *Gdf9*, *Pou5f1*, *Nobox* and *Kitl* [10]. In somatic tissues, Lhx8 plays an important role in the development of basal forebrain cholinergic neurons [12]. Lhx8 protein contains two LIM domains and one homeobox domain, which can promote protein interactions by regulating the activity of related molecules [13, 14].

Given the functional importance of Lhx8, understanding what proteins interact with it would provide new information for studying the mechanism of the regulatory roles of Lhx8 during oocyte and embryonic development. In a recent study, we have demonstrated that bovine Lhx8 interacts with Figla, a transcription factor essential for oogenesis [15]. In this study, we report the characterization of two rainbow trout *lhx8* genes, *lhx8a* and *lhx8b*, and the discovery of a novel germ cell-specific nuclear protein, Borealin 2, which interacts with Lhx8 proteins. Results indicate that both Lhx8a and Lhx8b function through interaction with Borealin-2, which may play an important role in oocyte and embryonic development in rainbow trout.

## Results

### Identification and expression analysis of rainbow trout *Lhx8* genes

Through searching a rainbow trout oocyte transcriptome database and the genome sequence [16], we identified two distinctive *lhx8* genes (named *lhx8*a and *lhx8b*). They are located on different chromosomes, with *lhx8a* being on chromosome 28 and *lhx8b* being on chromosome 8. The *lhx8*a cDNA sequence (1,956 bp) contains an open reading frame (ORF) of 1,035 bp encoding a protein of 344 amino acids. The *lhx8b* cDNA (1,373 bp) contains an ORF of 1,086 bp encoding a protein of 361 amino acids. Both proteins contain two LIM domains and one homeobox domain. They share 83% sequence identity and the sequence differences between the two proteins are mainly located at the C terminus (S1 Fig). The LIM and homeobox domains are very well conserved between two proteins, suggesting that both proteins are functional.

**Fig 1:**
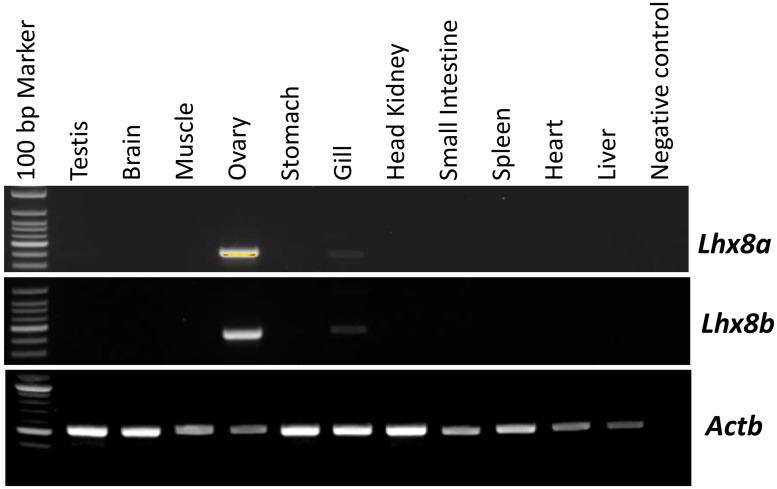
Tissue distribution analysis of *lhx8a* and *lhx8b* mRNA by RT-PCR. Trout tissues tested include testis, brain, muscle, ovary, stomach, gill, head kidney, small intestine, spleen, heart, and liver. Trout *actb* gene was used as a control for RNA quality.

**Fig 2:**
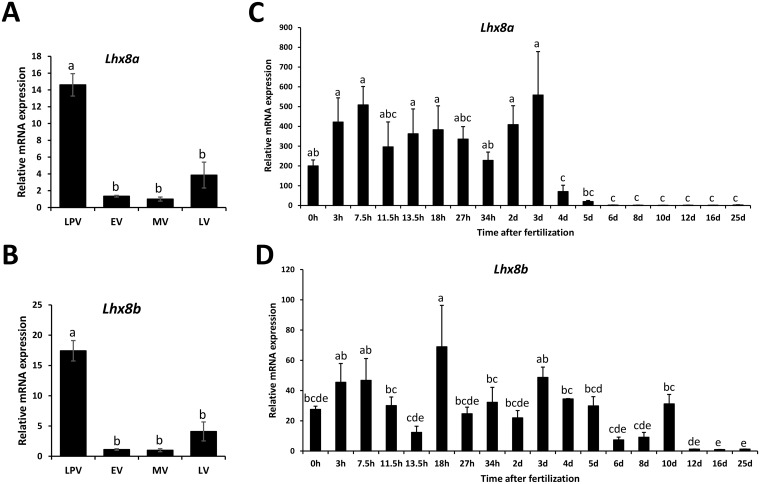
RT-qPCR analysis of *lhx8a* and *lhx8b* mRNA expression during ovarian development and embryogenesis. **A** and **B**: Expression of *lhx8a* and *lhx8b* mRNA during ovarian development. Ovarian samples (n = 4) at different developmental stages including late pre- (LPV), early- (EV), mid- (MV), and late-vitellogenesis (LV) were used in the analysis. The quantity of *lhx8a* and *lhx8b* mRNA was normalized to the quantity of *actb* gene. **C** and **D**: Expression of *lhx8a* and *lhx8b* mRNA during embryonic development. Embryos (n = 4) were collected at 0, 3, 7.5, 11.5, 13.5, 18, 27, 34 hours (h), 2, 3, 4, 5, 6, 8, 10, 12, 16, and 25 days (d) post-fertilization. The quantity of *lhx8a* and *lhx8b* mRNA was normalized to the quantity of *18S rRNA* gene. Means of the normalized gene expression values for each stage (ovarian or embryonic development) were calculated and expressed as relative fold changes. Different letters indicate significant difference (P<0.05).

Tissue distribution analysis of *lhx8a* and *lhx8b* mRNA by RT-PCR revealed that both transcripts are predominantly expressed in the ovary but barely detectable or undetectable in other tissues (Fig 1). To further analyze the expression of *lhx8a* and *lhx8b* during ovarian and embryonic development, we performed quantitative real-time PCR (RT-qPCR) analysis using samples collected from different stages of vitellogenesis and embryogenesis. Similar expression profiles of *lhx8a* and *lhx8b* during vitellogenesis were observed. Both genes show higher expression in ovaries containing only pre-vitellogenic follicles relative to more mature ovaries (Fig 2A and 2B). During early embryogenesis, the expression of *lhx8a* mRNA remains high in early stage embryos until 3-day embryos and decreases sharply in 4-day and 5-day embryos, eventually reaching an undetectable level in late stage embryos (6-day to 25-day embryos) (Fig 2C). The expression of *lhx8b* mRNA remains more or less consistent up to 10-day embryos and then decreases to an extremely low level in 12-day to 25-day embryos (Fig 2D).

### Interactions between Lhx8a and Lhx8b

**Fig 3:**
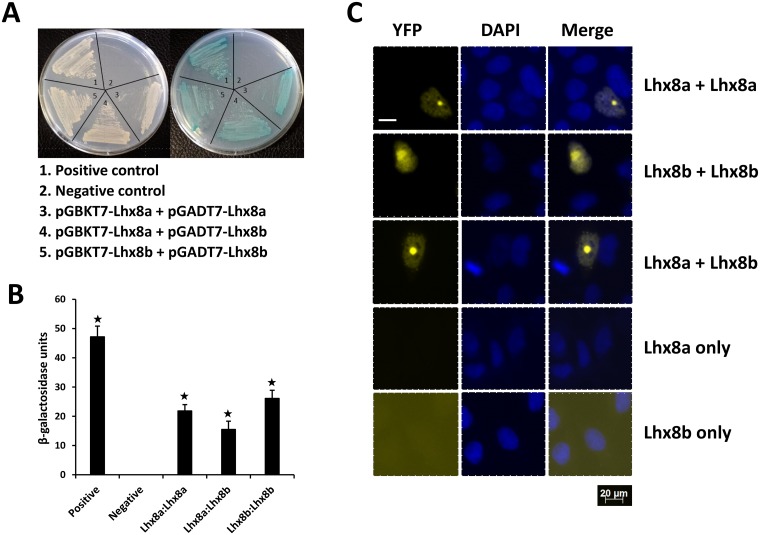
Analysis of interactions between Lhx8a and Lhx8b. **A:** Yeast two-hybrid analysis showing growth of yeast cells expressing BD and AD fusion proteins. Left plate: growth of yeast cells on DDO plate (medium lacking Leucine and tryptophan) showing successful co-transformations. Right plate: growth of yeast cells on selective QDO/X/A plate (quadruple drop-out medium lacking leucine, tryptophan, adenine and histidine and containing X-gal and Aureobasidin A) indicating protein-protein interactions. **B:** Yeast β-galactosidase liquid assays confirming protein-protein interactions. Asterisks indicate significant difference (P<0.05). **C**: BiFC analysis of protein-protein interactions. HEK293 cells were co-transfected with equal amounts of BiFC expression constructs encoding VN-Lhx8a and VC-Lhx8a, or VN-Lhx8b and VC-Lhx8b, or VN-Lhx8a and VC-Lhx8b fusion proteins. Single transfections using constructs encoding either VN-Lhx8a or VN-Lhx8b were served as negative controls. YFP signals due to BiFC were measured by fluorescence microscopy. Scale bars, 20 μm.

LIM domain is a unique double-zinc finger motif, which is believed to function as a dimerization domain [17]. To determine if Lhx8a and Lhx8b can form homodimer or heterodimer through their LIM domains, we performed yeast-two hybrid assays to access the interactions between Lhx8a and Lhx8b. The assays demonstrated that Lhx8a and Lhx8b not only can interact with each other, but also interact with themselves (Fig 3A). Further evaluation of the binding affinity of protein-protein interactions by β-galactosidase assays showed significantly higher β-galactosidase activities in yeast cells expressing the interacting partners relative to the control cells (Fig 3B). To verify the interactions between Lhx8a and Lhx8b observed by yeast-two hybrid assays, we performed bimolecular fluorescence complementation (BiFC) assays to visualize protein interactions in living cells. Mammalian expression plasmids encoding Lhx8a or Lhx8b fused to the N-terminal fragment (VN) of yellow fluorescent protein (YFP), or the C-terminal fragment (VC) of YFP, were generated and transfected into HEK293 cells. Fluorescence microscopy was applied to determine the BiFC efficiency of paired VN and VC fused Lhx8 proteins. Yellow fluorescent signals were observed in cells expressing Lhx8a-VN and Lhx8a-VC, Lhx8b-VN and Lhx8b-VC, and Lhx8a-VN and Lhx8b-VC (Fig 3C). BiFC signals of these protein complexes are localized in the nucleus, as seen by overlap with DAPI staining. It appears that the fluorescent signals are enriched in a single large subnuclear focus. No BiFC signals were detected in single transfection with either Lhx8a-VN or Lhx8b-VN constructs. Collectively, the results indicate that Lhx8a and Lhx8b may form heterodimer or homodimer.

### Identification of Borealin-2 as an Lhx8-interacting protein

**Fig 4:**
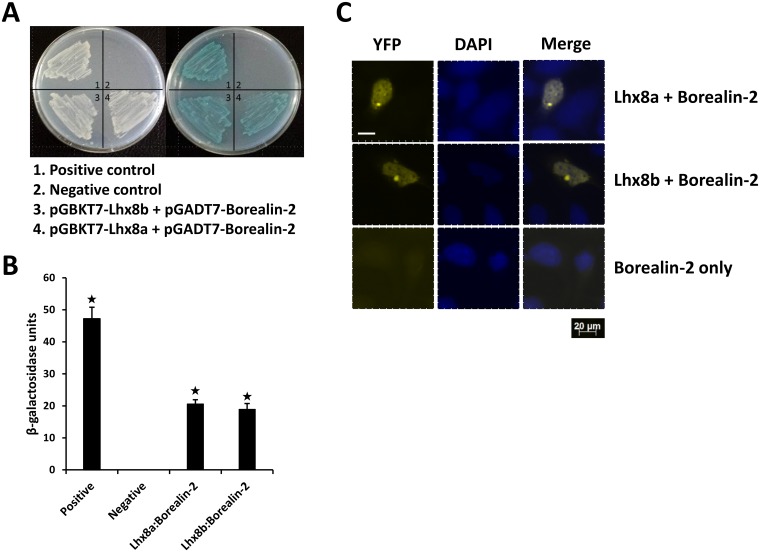
Analysis of interactions between Lhx8 proteins and Borealin-2. **A:** Yeast two-hybrid analysis showing growth of yeast cells expressing BD and AD fusion proteins. Left plate: growth of yeast cells on DDO plate (medium lacking Leucine and tryptophan) showing successful co-transformations. Right plate: growth of yeast cells on selective QDO/X/A plate (quadruple drop-out medium lacking leucine, tryptophan, adenine and histidine and containing X-gal and Aureobasidin A) indicating protein-protein interactions. **B:** Yeast β-galactosidase liquid assays confirming interactions between Lhx8 proteins and Borealin-2. Asterisks indicate significant difference (P<0.05). **C**: BiFC analysis showing interactions between Lhx8 proteins and Borealin-2. HEK293 cells were co-transfected with equal amounts of BiFC expression constructs encoding VN-Lhx8a and VC-Borealin-2, or VN-Lhx8b and VC-Borealin-2 fusion proteins. Single transfection using the construct encoding VC-Borealin-2 was served as a negative control. YFP signals due to BiFC were measured by fluorescence microscopy. Scale bars, 20 μm.

To identify cellular proteins that might interact with Lhx8 protein, we performed a yeast two-hybrid screening using Lhx8b as a bait. Two identical cDNA clones from a rainbow trout oocyte cDNA library were identified. Sequence analysis of the isolated clones revealed that they code for a novel protein of 255 amino acids containing an Nbl1 (Borealin_N) domain (GenBank accession number: CDQ55984.1). The novel protein is named as Borealin-2. The interactions between Borealin-2 and Lhx8a or Lhx8b were confirmed by retransformation of Borealin-2-AD fusion construct into Y187 yeast cells followed by mating with the Y2Gold cells expressing either Lhx8a-BD or Lhx8b-BD fusion proteins (Fig 4A). Further confirmation of the interactions between Borealin-2 and Lhx8a or Lhx8b were performed by β-galactosidase (Fig 4B) and BiFC assays (Fig 4C).

**Fig 5:**
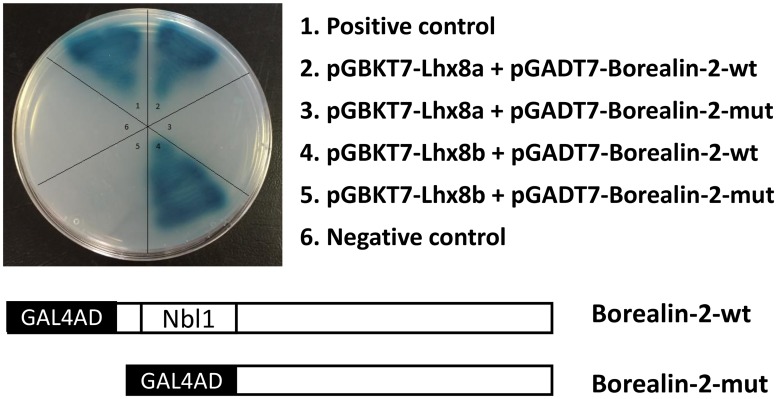
Requirement of the Nbl1 domain of Borealin-2 for interaction with Lhx8 proteins. A DNA fragment encoding Borealin-2 with its Nbl1 domain (amino acid position 28–80) deleted was amplified by PCR using gene-specific primers (Table 1) and cloned into pGADT7 vector, generating a mutant construct, pGADT7-Borealin-2-mut. Interactions between Lhx8 proteins (pGBKT7-Lhx8a or pGBKT7-Lhx8b) and wild type Borealin-2 (pGADT7-Borealin-2-wt) or mutant Borealin-2 (pGADT7-Borealin-2-mut) were assayed by direct yeast two-hybrid analysis. Yeast cells expressing BD and AD fusion proteins were grown on a QDO/X/A plate.

To determine if the Nbl1 domain is responsible for interaction with Lhx8 proteins, we performed a yeast two-hybrid assay using a mutant construct expressing Borealin-2 with its Nbl1 domain deleted. Blue colonies were readily detectable within three days when yeast cells expressing the wild type Borealin-2 was mated with yeast cells expressing either BD-fused Lhx8a or Lhx8b. However, only a few light blue colonies appeared after five days when cells expressing the mutant Borealin-2 was mated with cells expressing either BD-Lhx8a or BD-Lhx8b. Growth of yeast cells on a selective plate was observed when cells expressing the wild type Borealin-2 but not the mutant Borealin-2 (Nbl1 deletion) was mated with yeast cells expressing the Lhx8a or Lhx8b fusion proteins, indicating that the Nbl1 domain of Borealin-2 is required for interaction with Lhx8 proteins in vitro (Fig 5).

**Fig 6:**
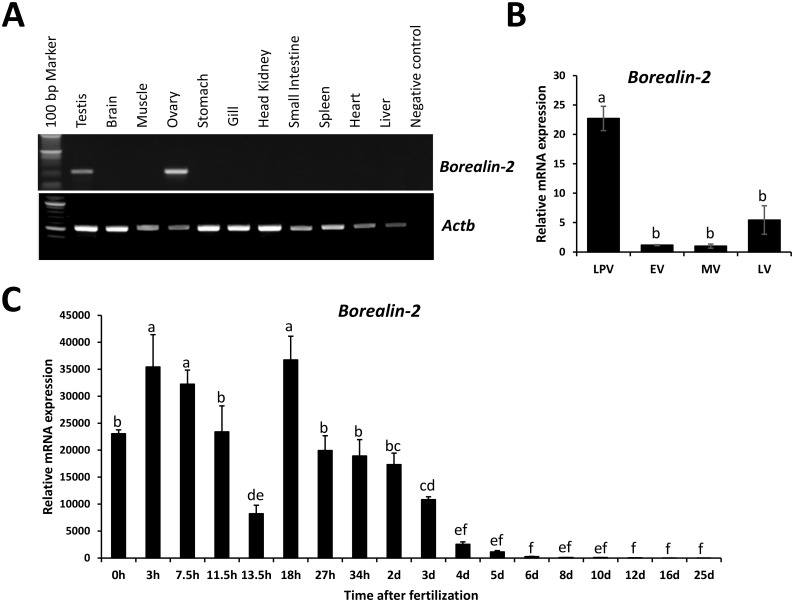
Analysis of *borealin-2* mRNA expression. **A**: RT-PCR analysis of *borealin-2* mRNA expression in rainbow trout tissues. Trout tissues tested include testis, brain, muscle, ovary, stomach, gill, head kidney, small intestine, spleen, heart, and liver. Trout *actb* gene was used as a control for RNA quality. **B**: RT-qPCR analysis of *borealin-2* mRNA expression during ovarian development. Ovarian samples (n = 4) at different developmental stages including late pre- (LPV), early- (EV), mid- (MV), and late-vitellogenesis (LV) were used in the analysis. The quantity of *borealin-2* mRNA was normalized to the quantity of *actb* gene. **C**: RT-qPCR analysis of *borealin-2* mRNA expression during embryonic development. Embryos (n = 4) were collected at 0, 3, 7.5, 11.5, 13.5, 18, 27, 34 hours (h), 2, 3, 4, 5, 6, 8, 10, 12, 16, and 25 days (d) post-fertilization. The quantity of *borealin-2* mRNA was normalized to the quantity of *18S rRNA* gene. Means of the normalized gene expression values for each stage (ovarian or embryonic development) were calculated and expressed as relative fold changes. Different letters indicate significant difference (P<0.05).

*borealin-2* is a novel gene that has not been characterized yet in rainbow trout. It was named after Borealin (also known as cell division cycle-associated protein 8 or Dasra-B), which contains the Nbl1 at the N terminus and a Borealin domain at the C terminus (S2 Fig). Through database mining, we identified Borealin-2 orthologs in other species including chicken (NP_001263267.1), X. laevis (NP_001086415.1), and zebrafish (NP_001189357.1) (S3 Fig). The Nbl1 domain is conserved among these species. Tissue distribution analysis revealed that the expression of rainbow trout *borealin-2* mRNA is restricted to the ovary and testis (Fig 6A). RT-qPCR analysis showed that expression of *borealin-2* mRNA is higher in ovaries in ovaries containing only pre-vitellogenic follicles relative to more mature ovaries (Fig 6B). The expression of *borealin-2* mRNA is also high in embryos at early stages and significantly decreases in 4-day embryos and progressively becomes undetectable (Fig 6C).

**Fig 7:**
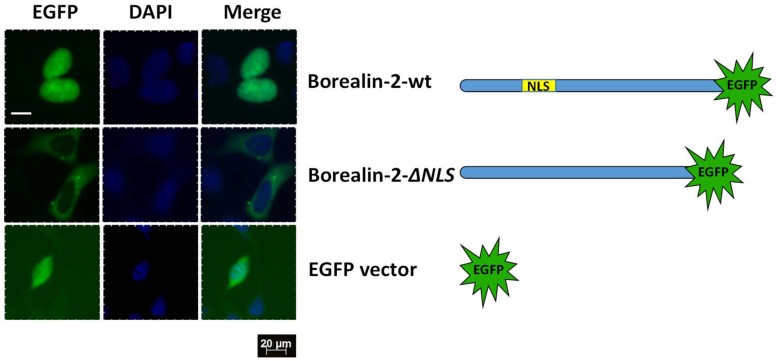
GFP reporter assay showing nuclear localization of Borealin-2 protein. HEK293 cells were transfected with GFP reporter constructs expressing either an EGFP-tagged wild type Borealin-2 (Borealin-2-wt) or a mutant Borealin-2 with its NLS deleted (Borealin-2-ΔNLS). Empty pcDNA3-EGFP vector was used as a control. Nuclear DNA was stained with DAPI and cells were analyzed with a fluorescence microscope. Scale bars, 20 μm.

Borealin-2 was predicted to possess a typical nuclear localization signal (NLS) indicating that it is a nuclear factor. To determine if Borealin-2 is a nuclear protein and if the predicted NLS is required for its nuclear localization, we performed a GFP reporter assay. HEK293 cells were transfected with expression constructs expressing either an EGFP-tagged wild-type Borealin-2 or a mutant Borealin-2 lacking the NLS. Fig 7 shows that the wild-type Borealin-2 is exclusively localized to the nucleus of the transfected cells, while the mutant Borealin-2 is enriched in the cytoplasm of the cells. The result confirms that Borealin-2 is a nuclear protein and the predicted NLS is required for its nuclear localization.

## Discussion

LIM homeodomain genes play vital roles in tissue patterning and differentiation [12]. Lhx8 represents the first LIM homeodomain family member that plays a crucial role in oogenesis [4]. In this study, we characterized the rainbow trout *lhx8* genes and discovered a novel oocyte-specific nuclear protein that interacts with Lhx8 proteins.

In rainbow trout, we identified 2 *lhx8* genes, *lhx8a* and *lhx8b*, which are located on two different chromosomes. The existence of two distinct *lhx8* genes in this species is most likely a result of genome duplication event [18]. The functional domains (LIM and homeobox) are very well conserved between the two proteins, indicating that Lhx8a and Lhx8b are functionally similar. Both *lhx8a* and *lhx8b* transcripts are predominantly expressed in the ovary, which is consistent with the findings in mouse [10], chicken [19] and pig [20].

The LIM domain is a protein-protein interaction interface [21] and is believed to function in protein dimerization [17]. Lhx8 contains two LIM domains with each having two zinc fingers. Results from the interaction assays show that Lhx8a and Lhx8b can interact with each other or themselves, indicating that the two proteins may form homodimers or heterodimers. Dimer formation not only influences the stability of the proteins but also increases the specificity and affinity of transcription factors binding to DNA domains.

Protein-protein interactions are critical in many biological and developmental processes. For example, decreased functional interaction between Figla and Tcf3 in human could cause premature ovarian failure [22]. A mini interactome centered by Nanog is required to maintain ES cell pluripotency [23]. In mouse, Lhx8 and Isl1 interaction is required for cholinergic gene expression in embryonic stem cell derived neurons [24]. In a previous study, we have shown that Lhx8 interacts with Figla, suggesting that these two germ cell-specific proteins may function in a cooperative fashion [15]. In the present study, we identified Borealin-2, a novel nuclear protein that interacts with both Lhx8a and Lhx8b through a yeast two-hybrid screening. Borealin-2 is considered as a paralogue of Borealin, which is a subunit of the chromosomal passenger complex (CPC) required for stability of the bipolar mitotic spindle [25] and plays a crucial role in early embryonic development in mouse [26, 27]. However, Borealin-2 has never been studied experimentally. It was named after Borealin as it contains an Nbl1 (or Borealin_N) domain. However, besides the Nbl1 domain, the rest of Borealin-2 sequence does not share significant sequence homology with Borealin. In addition, EST profiles (NCBI Unigene database) of human and mouse *Borealin* mRNA indicate that *Borealin* is expressed ubiquitously, while our data indicate that *borealin-2* expression is restricted to ovary and testis. The exact functions of Borealin-2 in gonads remain to be investigated. Interestingly, Borealin-2 has orthologs in chicken, X. laevis, and zebrafish but not in any mammalian species.

We provided evidence showing that *borealin-2* mRNA is specifically expressed in ovary and testis and the expression of *borealin-2* mRNA is developmentally regulated during vitellogenesis and early embryogenesis. We also demonstrated that Borealin-2 protein is localized in the nucleus and deletion of the Nbl1 domain leads to dramatically decreased interaction between Borealin-2 and Lhx8a or Lhx8b, indicating the Nbl1 domain is responsible for protein: protein interaction. As a germ cell-specific nuclear protein abundantly present in oocyte and early embryos, Borealin-2 may play a crucial role in oocyte and early embryonic development through interactions with Lhx8a and Lhx8b.

In summary, the present study characterized the rainbow trout *lhx8* genes and identified Borealin-2 as a novel germ cell-specific nuclear factor that interacts with rainbow trout Lhx8 proteins. The novel protein deserves further investigation with respect to its influence on the development of oocytes and early embryos in fish as well as in other species.

## Materials and methods

### Collection of fish samples

Mature eggs collected from three female fish spawns at the National Center for Cool and Cold Water Aquaculture (Kearneysville, WV) were artificially fertilized and raised in a flow-through system at 13.8°c. Embryonic samples were collected at specific time points after fertilization that include 0, 3, 7.5, 11.5, 13.5, 18, 27, 34 hours, 2, 3, 4, 5, 6, 8, 10, 12, 16 and 25 days post-fertilization. Different tissue samples including testis, brain, muscle, ovary, stomach, gill, head kidney, small intestine, spleen, heart and liver were collected from adult fish. Ovarian samples were collected at different reproductive stages: late pre-vitellogenesis (follicle ø under ~0.65 mm), early vitellogenesis (follicles ø up to ~0.65–1.1 mm), mid-vitellogenesis (follicles ø up to 1.1–2.1 mm) and late vitellogenesis (follicles ø up to ~2.1–4.0 mm)[6]. All samples were quick-frozen in liquid nitrogen, and stored in -80°C until use. All experiments were conducted under a protocol approved by the West Virginia University Institutional Animal Care and Use Committee (protocol #13–0601).

### Reverse transcription polymerase chain reaction (RT-PCR)

Total RNA from different tissues was isolated using Trizol reagent according to the manufacturer’s instructions (Invitrogen, Carlsbad, CA). After DNase I treatment, the RNA was converted to cDNA using oligo (dT)_18_ primer and SuperScript III reverse transcriptase (Invitrogen, Carlsbad, CA). RT-PCR was performed in a 25-μl reaction using gene specific primers (Table 1) under the following conditions: 5 min denaturation at 94°C followed by 30 cycles of 94°C for 30 sec, 58°C for 45 sec, and 72°C for 30 sec, and a final extension at 72°C for 10 min. Rainbow trout β-actin gene (*actb*) was used as a control for RNA quantity.

**Table 1:** Primers used in this study.

Primer name	Sequence	Application
Lhx8a-RTPCR-F	CAGGCCATGGAAAGTGGTGTTAC	RT-PCR
Lhx8a-RTPCR-R	GAGGGAGAGTGCACATCCATG	RT-PCR
Lhx8b-RTPCR-F	GTTAGAGCAAGAAGGGTCCCAGG	RT-PCR
Lhx8b-RTPCR-R	CTGTGATCTCCAAGTGCCGACT	RT-PCR
Borealin-2-RTPCR-F	GTGAGTGAAGGTGCATCGGCTC	RT-PCR
Borealin-2-RTPCR-R	CCAGGCAGGGAGCAATACAGG	RT-PCR
Actb-RT-PCR-F911	AAGTGTGACGTGGACATCCGT	RT-PCR
Actb-RT-PCR-R1421	TAATCCGCTGCTTCACCGTTC	RT-PCR
Lhx8a-realtime-F	ACAGCAGTCCAGGCTTTCTC	Real-time PCR
Lhx8a-realtime-R	GAGGGAGAGTGCACATCCATG	Real-time PCR
Lhx8b-realtime-F	ACAGACACACCCATGCACAC	Real-time PCR
Lhx8b-realtime-R	CTGTGATCTCCAAGTGCCGACT	Real-time PCR
Actb-realtime-F541	GCCGGCCGCGACCTCACAGACTAC	Real-time PCR
Actb-realtime-R613	CGGCCGTCCTCCTGAAGCTGTAAC	Real-time PCR
18S rRNA-realtime-F	CATGGCCGTTCTTAGTTGGT	Real-time PCR
18S rRNA-realtime-R	CTCTAAGAAGTTGGACGCCG	Real-time PCR
Lhx8a/b-AD/BD-F	GGCCCATATGTATTGGAAAAGTGAACTAATG	Yeast two-hybrid
Lhx8a/b-AD/BD-R	GGCCGAATTCGGCATGGCTGATTGGCAGCTGT	Yeast two-hybrid
Borealin-2-mut-AD-F	GGCCCATATG GCGGATGACATTTCTGCAGGTG	Yeast two-hybrid NBl1 del
Borealin-2-mut-AD-R	GGCCGAATTC TCATTGCACACTCAGAGGCTG	Yeast two-hybrid NBl1 del
Lhx8a-BiFC-F	GGCCGAATTCGATGTATTGGAAAAGTGAACTAATG	BiFC
Lhx8a-BiFC-R	GGCCTCTAGAGGCATGGCTGATTGGCAGCTGT	BiFC
Lhx8b-BiFC-F	GGCCGAATTCGGATGGATTGGAAAAGTGAACTAATG	BiFC
Lhx8b-BiFC-R	GGCCCTCGAGCGGCATGGCTGATTGGCAGCTGT	BiFC
Borealin-2-BiFC-F	GGCCGAATTCGGATGGCACCAAGAAGGATAAGG	BiFC
Borealin-2-BiFC-R	GGCCCTCGAGCTTGCACACTCAGAGGCTG	BiFC
Borealin-2-EGFP-F	GGCCGAATTCATGGCACCAAGAAGGATAAGG	EGFP assay
Borealin-2-EGFP-R	GGCCCTCGAGTTGCACACTCAGAGGCTGATTCGC	EGFP assay
Borealin-2-delNLS-R	AGCCGATGCACCTTCACTCAC	EGFP assay NLS del
Borealin-2-delNLS-F	GTGAGTGAAGGTGCATCGGCTGCCAATAGCTCCAGCACTGGAAGCC	EGFP assay NLS del

### Quantitative real time polymerase chain reaction (RT-qPCR)

Total RNA from ovaries and embryos was isolated using Trizol reagent. RNA isolated from embryos was further purified using lithium chloride precipitation. Following DNase I treatment, the RNA was converted to cDNA using a mixture of oligo (dT)_18_ primer and random hexamers, and SuperScript III reverse transcriptase (Invitrogen, Carlsbad, CA). RT-qPCR was performed on a Bio-Rad CFX96^™^ Real-Time PCR Detection System using iQ^™^ SYBR Green Supermix (Bio-Rad, Hercules, CA) in a 10-μl reaction. Rainbow trout *actb* or *18S rRNA* gene was used as endogenous controls. The primers used in RT-qPCR analysis are listed in Table 1. Cycling parameters were set as 95°c for 3 min followed by 40 cycles of 95°c for 10 sec and 60°c for 1 min. Melting-curve analyses were included after the amplification. Standard curves for each gene and the endogenous controls were constructed using serial dilutions of a pooled cDNA sample. The quantity of the target genes and the endogenous control genes in each sample was determined from respective standard curves. The quantity of the target gene mRNA was then normalized to the quantity of *actb* mRNA (for expression in ovaries) or *18S rRNA*) (for expression in embryos). One-way analysis of variance (ANOVA) followed by multiple comparison using Student's t-tests was performed on normalized gene expression values using JMP (SAS Institute, Cary, NC).

### Yeast two-hybridization

Yeast two-hybridization is a commonly used molecular biology technique for studying protein-protein interactions [28]. In this study, the Matchmaker Two-Hybrid System (Clontech Laboratories, Mountain View, CA) was used to evaluate interactions between Lhx8a and Lhx8b, and identify proteins that interact with Lhx8 proteins. Interactions between Lhx8a and Lhx8b was assayed by direct yeast two-hybrid analysis. The coding regions of *lhx8a* and *lhx8b* were cloned in-frame into pGBKT7 or pGADT7 vector. The pGBKT7 constructs (pGBKT7-Lhx8a or pGBKT7-Lhx8b) were transformed into Y2HGold strain cells and the pGADT7 constructs (pGADT7-Lhx8a or pGADT7-Lhx8b) were transformed into Y187 strain cells. All transformants were tested for toxicity and auto-activation before mating according to the manufacturer's protocol. After mating for 24 h at 30°c, yeast cells were plated on synthetic double dropout selection medium DDO/X/A and incubated at 30°c for 3 days. Single blue colonies (>2mm) were selected and streaked onto fresh QDO/X/A quadruple dropout plates (lacking adenine, histidine, tryptophan and leucine and supplemented with X-α-Gal and Aureobasidin A).

To identify Lhx8 interacting proteins, a rainbow trout cDNA library constructed in pGADT7 plasmid using mature unfertilized oocytes was screened with the bait expression plasmid, pGBKT7-Lhx8b. Yeast cells harboring the cDNA library were mated with Y2HGold yeast cells containing pGBKT7-Lhx8b plasmid for 24 h at 30°c, followed by plating and incubation on DDO/X/A plates for 3–5 days. Blue colonies were re-streaked on QDO/X/A plates for high stringent selection. Plasmids were isolated from blue colonies grown on QDO/X/A plates, and then transformed into competent *E*. *coli* cells using carbenicillin to select for pGADT7-resistant clones. The plasmids from the resistant clones were sequenced and the sequences were used to BLAST against the GenBank database.

To further confirm the screening results, the selected plasmid was re-transformed into the host strain, directly mated with yeast cells harboring the bait plasmid for 24 h and then plated onto QDO/X/A plates. The transformants were tested for β-galactosidase activity by yeast β-galactosidase liquid assay as described previously [9].

### GFP reporter assay

The coding region of rainbow trout wild-type *borealin-2* was PCR-amplified using gene-specific primers containing NdeI and EcoRI sites (Table 1) and cloned in-frame with the EGFP sequence in pcDNA3-EGFP vector (Addgene, Cambridge, MA) to generate the expression plasmid, pcDNA3-EGFP-Borealin-2-wt. The mutant Borealin-2 was generated by 2-step PCR using primers designed to produce a mutant with the NLS (amino acid position from 120 to 138) deleted (Table 1) and cloned in-frame with the EGFP sequence in pcDNA3-EGFP vector (pcDNA3-EGFP-Borealin-2-ΔNLS). Both constructs were confirmed by sequencing.

HEK293 cells were plated onto 20-mm diameter Poly-D-lysine-coated coverslips (Neuvitro, El Monte, CA) placed inside 6-well plates. Twenty-four hours after seeding, cells were transfected with constructs expressing either the EGFP-tagged wild-type Borealin-2 (pcDNA3-EGFP-Borealin-2-wt) or the mutant Borealin-2 (pcDNA-EGFP-Borealin-2-ΔNLS). Twenty-four hours after transfection, cells on coverslips were washed with 1 × PBS and fixed in methanol for 5 min followed by DAPI staining. Cells were analyzed using a fluorescence microscope (MIF Zeiss Fluorescent).

### Bimolecular fluorescence complementation (BiFC)

The BiFC plasmids, pBiFC-VN173 and pBiFC-VC155, were obtained from Addgene (Cambridge, MA). Coding regions of *lhx8a*, *lhx8b* and *borealin-2* were PCR-amplified and cloned into pBiFC-VN173 and pBiFC-VC155 vectors, generating pBiFC-VN173-Lhx8a, pBiFC-VC155-Lhx8a, pBiFC-VN173-Lhx8b, pBiFC-VC155-Lhx8b, and pBiFC-VC155-Borealin-2 fusion constructs. HEK293 cells were co-transfected with equal amounts of a VN and a VC fusion constructs. Single transfections using either pBiFC-VN173-Lhx8a or pBiFC-VN173-Lhx8b were served as negative controls. YFP signals due to BiFC were measured by fluorescence microscopy (MIF Zeiss Fluorescent).

## Supporting information

S1 Fig Comparison of protein sequences between Lhx8a and Lhx8b. Sequence alignment was performed using Clustal Omega (http://www.ebi.ac.uk/Tools/msa/clustalo/). The functional domains were determined by searching the Pfam database (http://pfam.xfam.org/search). The LIM and Homeobox domains are indicated by red and green boxes, respectively. The nuclear localization signals (NLS) were predicted using cNLS Mapper (http://nls-mapper.iab.keio.ac.jp/cgi-bin/NLS_Mapper_y.cgi) and they are indicated by asterisks.
(PDF)

S2 Fig Schematic representation of rainbow trout Borealin and Borealin-2 protein structures. (PDF)

S3 Fig Multiple sequence alignment of Borealin-2 proteins. Sequence alignment was performed using Clustal Omega (http://www.ebi.ac.uk/Tools/msa/clustalo/). The Nbl1 domain is indicated by a red box. RT-Borealin-2: rainbow trout Borealin-2 (CDQ55984.1), C-Borealin-2: chicken Borealin-2 (NP_001263267.1), Z-Borealin-2: zebrafish Borealin-2 (NP_001189357.1), X-Borealin-2: Xenopus laevis Borealin-2 (NP_001086415.1).
(PDF)
