# Supplementary material for: Lhx8 interacts with a novel germ cell-specific nuclear factor containing an Nbl1 domain in rainbow trout (Oncorhynchus mykiss)
Source: PLoS One. 2017 Feb 2;12(2):e0170760. doi: 10.1371/journal.pone.0170760 (PMC5289475; doi:10.1371/journal.pone.0170760)
Supplement: S1 Fig — Sequence alignment was performed using Clustal Omega (http://www.ebi.ac.uk/Tools/msa/clustalo/). The functional domains were determined by searching the Pfam database (http://pfam.xfam.org/search). The LIM and Homeobox domains are indicated by red and green boxes, respectively. The nuclear localization signals (NLS) were predicted using cNLS Mapper (http://nls-mapper.iab.keio.ac.jp/cgi-bin/NLS_Mapper_y.cgi) and they are indicated by asterisks. (PDF) [file pone.0170760.s001.pdf]

S1 Fig

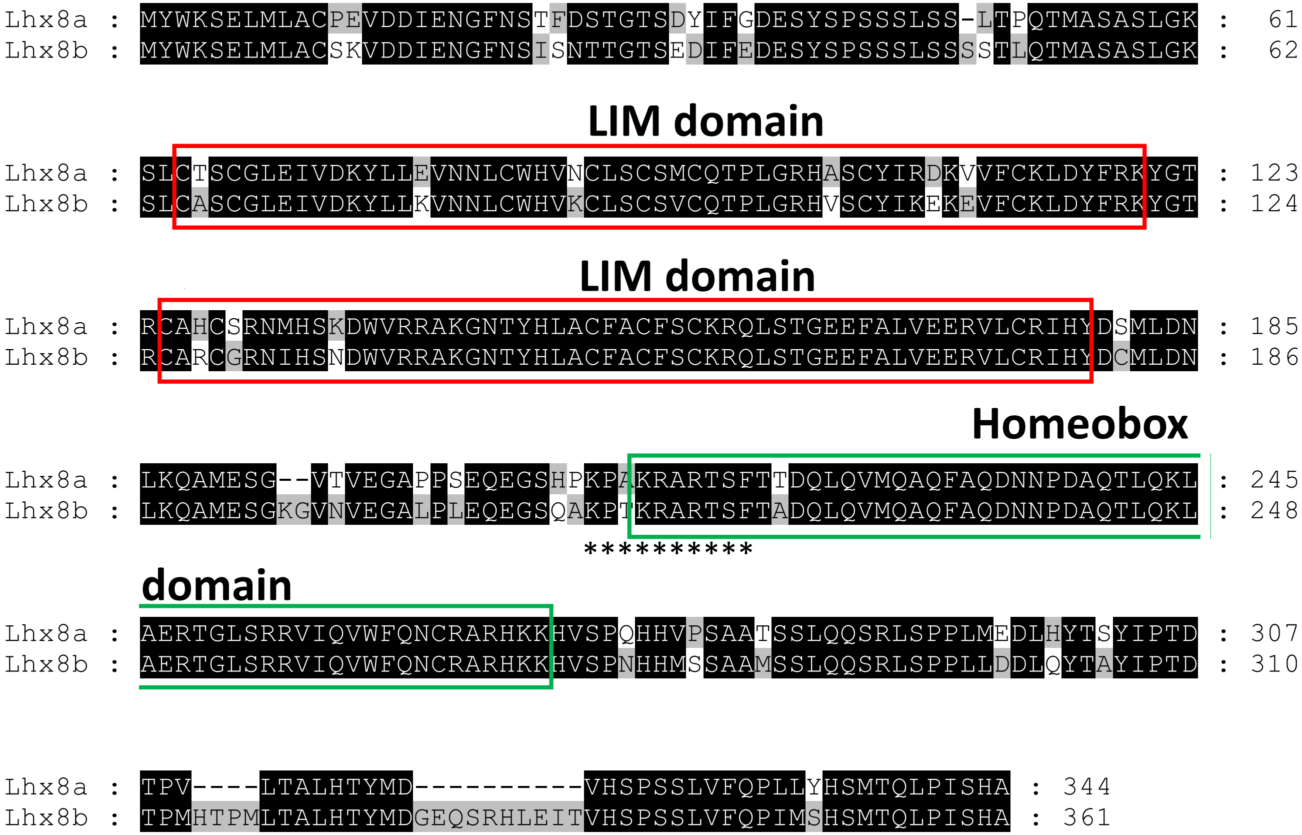
Lhx8a : MYWKSELMLACPEVDDIENGFNSTFDSTGTSDYIFGDESYSPSSSLSS-LTPQTMASASLGK : 61
Lhx8b : MYWKSELMLACSKVDDIENGFNSISNTTGTSEDIFEDESYSPSSSLSSSSTLQTMASASLGK : 62
LIM domain
Lhx8a : SLCTSCGLEIVDKYLLEVNNLCWHVNCLSCSMCQTPLGRHASCYIRDKVVFCKLDYFRKYGT : 123
Lhx8b : SLCASCGLEIVDKYLLKVNNLCWHVKCLSCSVCQTPLGRHVSCYIKEKEVFCKLDYFRKYGT : 124
LIM domain
Lhx8a : RCAHCSRNMHSKDWVRRAKGNTYHLACFACFSCKRQLSTGEEFALVEERVLCRIHYDSMLDN : 185
Lhx8b : RCARCGRNIHSNDWVRRAKGNTYHLACFACFSCKRQLSTGEEFALVEERVLCRIHYDCMLDN : 186
Homeobox
Lhx8a : LKQAMESG--VTVEGAPPSEQEGSHPKPAKRARTSFTTDQLQVMQAQFAQDNNPDAQTLQKL : 245
Lhx8b : LKQAMESGKGVNVEGALPLEQEGSQAKPTKRARTSFTADQLQVMQAQFAQDNNPDAQTLQKL : 248
****
domain
Lhx8a : AERTGLSRRVIQVWFQNCRARHKKHVSPQHHVPSAATSSLQQSRLSPPLMEDLHYTSYIPTD : 307
Lhx8b : AERTGLSRRVIQVWFQNCRARHKKHVSPNHHMSSAAMSSLQQSRLSPPLLDDLQYTAYIPTD : 310
Lhx8a : TPV----LTALHTYMD----------VHSPSSLVFQPLLYHSMTQLPISHA : 344
Lhx8b : TPMHTPMLTALHTYMDGEQSRHLEITVHSPSSLVFQPIMSHSMTQLPISHA : 361
