# Supplementary figures and images for: Lhx8 interacts with a novel germ cell-specific nuclear factor containing an Nbl1 domain in rainbow trout (Oncorhynchus mykiss)
Source: PLoS One. 2017 Feb 2;12(2):e0170760. doi: 10.1371/journal.pone.0170760 (PMC5289475; doi:10.1371/journal.pone.0170760)

S2 Fig

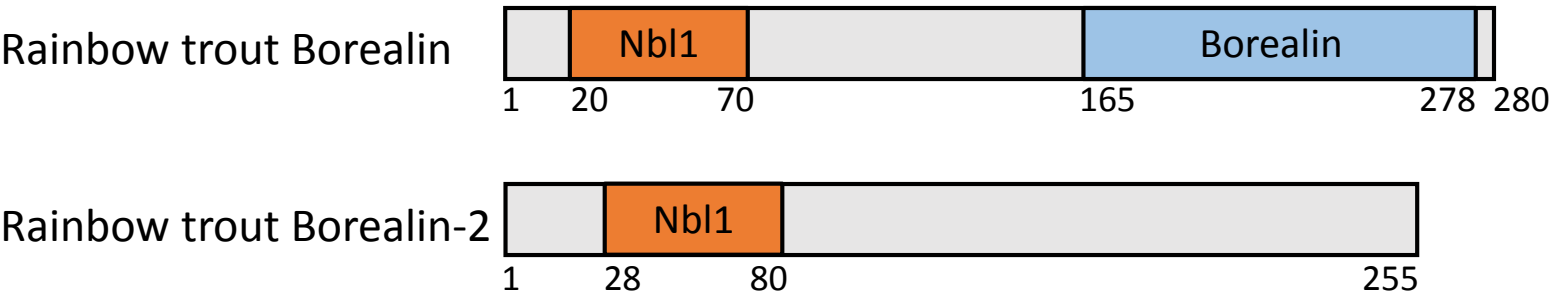

Supplement: S2 Fig — (PDF) [file pone.0170760.s002.pdf]
