# Supplementary material for: Lhx8 interacts with a novel germ cell-specific nuclear factor containing an Nbl1 domain in rainbow trout (Oncorhynchus mykiss)
Source: PLoS One. 2017 Feb 2;12(2):e0170760. doi: 10.1371/journal.pone.0170760 (PMC5289475; doi:10.1371/journal.pone.0170760)
Supplement: S3 Fig — Sequence alignment was performed using Clustal Omega (http://www.ebi.ac.uk/Tools/msa/clustalo/). The Nbl1 domain is indicated by a red box. RT-Borealin-2: rainbow trout Borealin-2 (CDQ55984.1), C-Borealin-2: chicken Borealin-2 (NP_001263267.1), Z-Borealin-2: zebrafish Borealin-2 (NP_001189357.1), X-Borealin-2: Xenopus laevis Borealin-2 (NP_001086415.1). (PDF) [file pone.0170760.s003.pdf]

S3 Fig

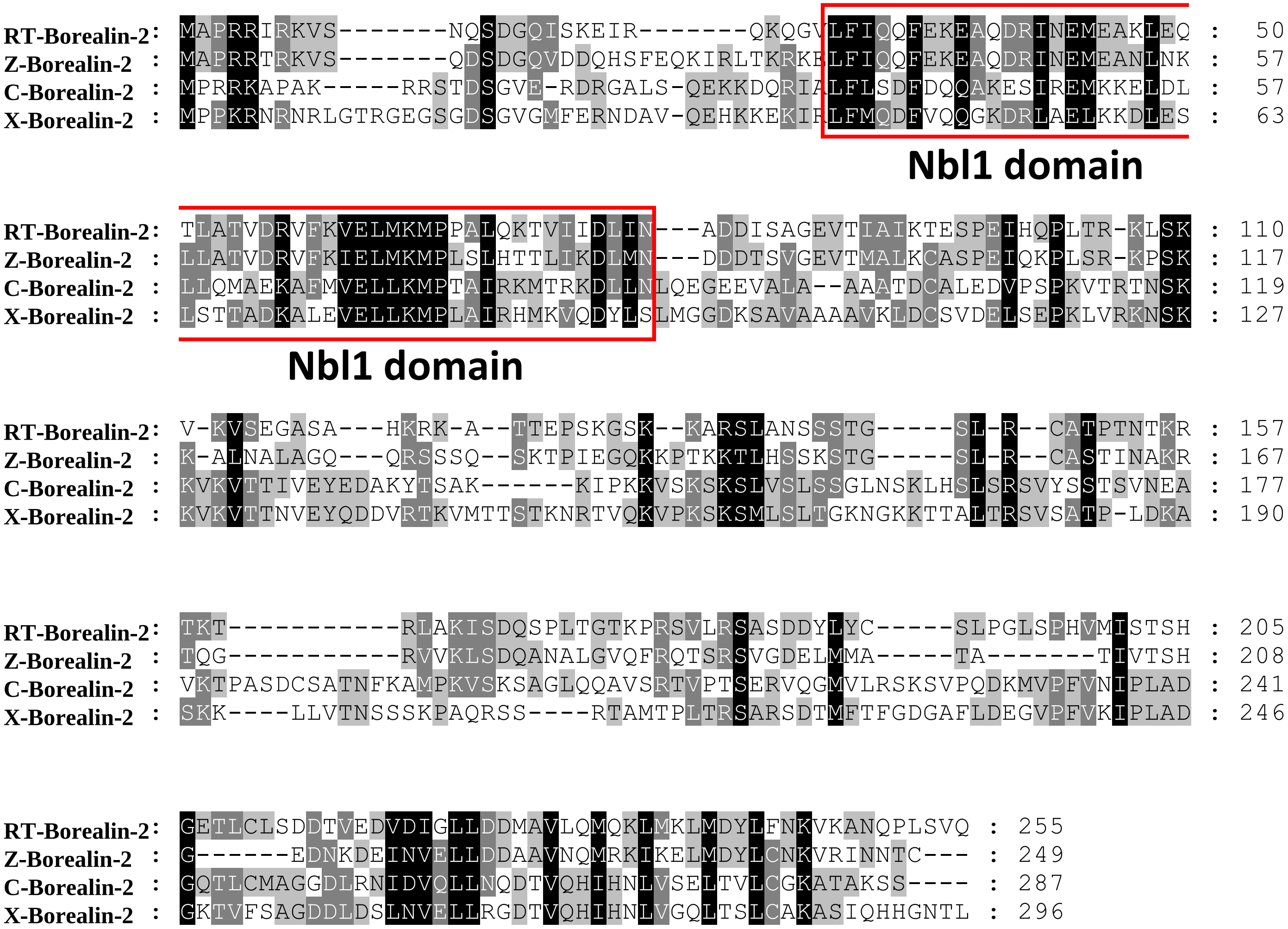
RT-Borealin-2 : MAPRRIRKVS-------NQSDGQISKEIR-------QKQGVLFIQQFEKEAQDRINEMEAKLEQ : 50
Z-Borealin-2 : MAPRRTRKVS-------QDSDGQVDDQHSFEQKIRLTKRKELFIQQFEKEAQDRINEMEANLNK : 57
C-Borealin-2 : MPRRKAPAK-----RRSTDSGVE-RDRGALS-QEKKDQRIALFLSDFDQQAKESIREMKKELDL : 57
X-Borealin-2 : MPPKRNRNRLGTRGEGSGDSGVGMFERNDAV-QEHKKEKIRLFMQDFVQQGKDRLAELKKDLES : 63
Nbl1 domain
RT-Borealin-2 : TLATVDRVFKVELMKMPPALQKTVIIDLIN---ADDISAGEVTIAIKTESPEIHQPLTR-KLSK : 110
Z-Borealin-2 : LLATVDRVFKIELMKMPLSLHTTLIKDLMN---DDDTSVGEVTMALKCASPEIQKPLSR-KPSK : 117
C-Borealin-2 : LLQMAEKAFMVELLKMPTAIRKMTRKDLLNLQEGEEVALA--AAATDCALEDVPSPKVTRTNSK : 119
X-Borealin-2 : LSTTADKALEVELLKMPLAIRHMKVQDYLSLMGGDKSAVAAAAVKLDCSVDELSEPKLVRKNSK : 127
Nbl1 domain
RT-Borealin-2 : V-KVSEGASA---HKRK-A--TTEPSKGSK--KARSLANSSSTG-----SL-R--CATPTNTKR : 157
Z-Borealin-2 : K-ALNALAGQ---QRSSSQ--SKTPIEGQKKPTKKTLHSSKSTG-----SL-R--CASTINAKR : 167
C-Borealin-2 : KVKVTTIVEYEDAKYTSAK------KIPKKVSKSKSLVSLSSGLNSKLHSLSRSVYSSTSVNEA : 177
X-Borealin-2 : KVKVTTNVEYQDDVRTKVMTTSTKNRTVQKVPKSKSMLSLTGKNGKKTTALTRSVSATP-LDKA : 190
RT-Borealin-2 : TKT-----------RLAKISDQSPLTGTKPRSVLRSASDDYLYC-----SLPGLSPHVMISTSH : 205
Z-Borealin-2 : TQG-----------RVVKLSDQANALGVQFRQTSRSVGDELMMA-----TA-------TIVTSH : 208
C-Borealin-2 : VKTPASDCSATNFKAMPKVSKSAGLQQAVSRTVPTSERVQGMVLRSKSVPQDKMVPFVNIPLAD : 241
X-Borealin-2 : SKK----LLVTNSSSKPAQRSS----RTAMTPLTRSARSDTMFTFGDGAFLDEGVPFVKIPLAD : 246
RT-Borealin-2 : GETLCLSDDTVEDVDIGLLDDMAVLQMQKLMKLMDYLFNKVKANQPLSVQ : 255
Z-Borealin-2 : G------EDNKDEINVELLDDAAVNQMRKIKELMDYLCNKVRINNTC--- : 249
C-Borealin-2 : GQTLCMAGGDLRNIDVQLLNQDTVQHIHNLVSELTVLCGKATAKSS---- : 287
X-Borealin-2 : GKTVFSAGDDLDSLNVELLRGDTVQHIHNLVGQLTSLCAKASIQHHGNTL : 296
